# Rapidly progressive glomerulonephritis in children

**DOI:** 10.12669/pjms.38.ICON-2022.5774

**Published:** 2022-01

**Authors:** Khemchand N Moorani, Madiha Aziz, Farhana Amanullah

**Affiliations:** 1 Khemchand N Moorani, MBBS, FCPS (Pediatrics and Nephrology). Professor of Pediatric Nephrology, The Kidney Center Postgraduate Training Institute (TKCPGTI), Karachi, Pakistan; 2 Madiha Aziz, MBBS, FCPS (Pediatrics and Nephrology). Pediatric Nephrologist, Sindh Institute of Urology & Transplantation, Karachi, Pakistan; 3 Farhana Amanullah, MBBS, DABP, FAAP. Pediatric Nephrologist, The Indus Hospital and Health Network, and the Aga Khan University Hospital, Karachi, Pakistan

**Keywords:** RPGN, Acute glomerulonephritis, Crescentic GN, Acute kidney injury, Immunosuppressive therapy

## Abstract

Rapidly progressive glomerulonephritis (RPGN), characterized by a rapid development of nephritis with loss of kidney function in days or weeks, is typically associated histologically, with crescents in most glomeruli; and is a challenging problem, particularly in low resource settings. RPGN is a diagnostic and therapeutic emergency requiring prompt evaluation and treatment to prevent poor outcomes. Histopathologically, RPGN consists of four major categories, anti-glomerular basement membrane (GBM) disease, immune complex mediated, pauci-immune disorders and idiopathic /overlap disorders. Clinical manifestations include gross hematuria, proteinuria, oliguria, hypertension and edema. Diagnostic evaluation, including renal function tests, electrolytes, urinalysis/microscopy and serology including (anti GBM antibody, antineutrophil cytoplasmic antibody (ANCA)) starts simultaneously with management. An urgent renal biopsy is required to allow specific pathologic diagnosis as well as to assess disease activity and chronicity to guide specific treatment.

The current guidelines for management of pediatric RPGN are adopted from adult experience and consist of induction and maintenance therapy. Aggressive combination immunosuppression has markedly improved outcomes, however, nephrotic syndrome, severe acute kidney injury requiring dialysis, presence of fibrous crescents and chronicity are predictors of poor renal survival. RPGN associated post infectious glomerulonephritis (PIGN) usually has good prognosis in children without immunosuppression whereas immune-complex-mediated GN and lupus nephritis (LN) are associated with poor prognosis with development of end stage kidney disease (ESKD) in more than 50% and 30% respectively.

Given the need for prompt diagnosis and urgent treatment to avoid devastating outcomes, we conducted a review of the latest evidence in RPGN management to help formulate clinical practice guidance for children in our setting.

***Information sources and search strategy:*** The search strategy was performed in the digital databases of PubMed, Cochrane Library, google scholar, from their inception dates to December 2020. Three investigators independently performed a systematic search using the following search terms “Rapidly progressive glomerulonephritis” “children” “crescentic glomerulonephritis” “management” at the same time, backtracking search for references of related literature.

## BACKGROUND

RPGN without prompt optimal management is a setup for morbidity and mortality.^1^ Different pathologic mechanisms can lead to crescent formation, inflammation and scarring and present as RPGN (also known as crescentic GN or cGN). When more than half the glomeruli are involved it leads to acute kidney injury (AKI) with more than 50% loss of renal function within a few days to weeks.^2^ The clinical severity varies from AKI to advanced uremia depending upon degree of glomerular involvement with crescents.^1^

## EPIDEMIOLOGY

The true incidence of RPGN is unknown due to its variable definitions and multiple underlying etiologies. The estimated incidence of RPGN is seven cases per million annually in the United States. It constitutes 3.2% and 5.5% of renal biopsies in adults from Saudi Arabia and India respectively.^3^,^4^ A study conducted at a major kidney diseases Institute in Karachi found that 46% (29/63) children with mesangiocapillary GN on biopsy presented with RPGN, indicating immune mediated GN as an important cause of RPGN in our setting. More than half the children with RPGN had poor outcomes despite treatment.^5^ It is important to understand thatcrescentric glomerulonephritis (cGN) is a pattern that can occur in a variety of glomerular diseases.^6^ A recent study that analyzed biopsies of 60 children with RPGN/cGN found immune complex GN in 75%, ANCA-associated pauci-immune GN in 17% and anti-glomerular basement-membrane GN in 2%. The same study found that cGN was detected in 7.5% (61/808) renal biopsies performed in children at their center.^6^ In children most RPGN is seen in the setting of immune complex (IC) mediated GN (PSGN, SLE, IgA, HSP).^7^,^8^ An Indian study showed a higher prevalence of pauci immune (PI) mediated RPGN compared to IC (71.7% vs 28.3%)^9^ raising the concern that PI is also an important cause of RPGN in children.^10^

## ETIOLOGY OF RAPIDLY PROGRESSIVE GLOMERULONEPHRITIS

The most appropriate classification of RPGN is based on histopathology and on the presence, localization, and characteristics of immune deposits on immunofluorescence (IF) staining and is divided into three major categories; Type-I: Linear antibody (IgG) deposition disorders: Anti-glomerular basement membrane (GBM) disease, Type-II: GN caused by deposition of immune complexes (i.e., in IgA nephropathy (IgAN), lupus nephritis (LN), PIGN, Henoch Schonlein Purpura nephritis (HSPN)) and Type-III: Pauci-immune GN (caused by ANCA vasculitis). Etiologies underlying RPGN are further listed in Table-I.

**Table I:** Etiology of RPGN (Crescentic GN) in Children.

**Anti-GBM linear (IgG deposits) GN**
Anti-GBM Nephritis
Goodpasture Syndrome
Post renal transplantation in Alport’s Syndrome
**Immune complex (granular deposits 80%) GN**
***Post infectious GN***
Post streptococcal GN
Infective endocarditis
Shunt nephritis
Staph aureus sepsis
Other infections: HIV, Hepatitis B and C
***Systemic disease***
SLE nephritis
HSP nephritis
Mixed connective tissue disorder
***Primary GN***
IgA nephropathy, MPGN, membranous nephropathy, C1q nephropathy
**Pauci-immune (negative IF or few deposits) GN**
Microscopic polyangitis
Granulomatosis with polyangitis (Wegener’s granulomatosis)
Renal limited vasculitis
Eosinophilic granulomatosis with polyangitis (Churg-Strauss disease)
Idiopathic crescentic GN
Medications: penicillamine, hydralazine
**Post-renal transplant (recurrence)**
IgAN, HSP, MPGN, SLE
**RPGN like clinical picture but without crescents**
Hemolytic uremic syndrome, acute interstitial nephritis, diffuse proliferative GN

Adapted from: Peadiatric kidney Disease - Chapter on RPGN 2016.

## PATHOGENESIS OF CRESCENTIC GLOMERULONEPHRITIS

Crescent formation represents a nonspecific response to severe injury to the glomerular capillary wall. There are three stages of crescent formation (Fig.1). During the first stage, there is induction of gaps or rents in the glomerular capillary wall and GBM by macrophages and T-cells. This is followed by movement of plasma proteins (fibrinogen) and inflammatory cells which release interleukin-1 (IL1) and tumor necrosis factor alfa (TNF alfa) and pro-coagulant factors into the bowman’s space.^11^,^12^ Crescents are the final common pathway of severe inflammatory glomerular disease. These include immune complex disease (like LN, IgA nephropathy, post infectious GN (PIGN)) as well as anti-GBM or ANCA related disorders.

The second stage is of active proliferative inflammation resulting in the development of cellular, fibrocellular and then fibrous crescents with the passage of time. Fibroblast growth factors promote fibroblast proliferation and collagen deposition. Transforming growth factor-beta may also play an important role. Apart from macrophages and T-cells, parietal and visceral epithelial cells also contribute in active crescent formation. This final fibrous stage is unlikely to respond to immunosuppressive therapy and has a high risk of ESKD.^1^,^13^,^14^

### Types of Crescentic Glomerulonephritis:

Three mechanisms of glomerular injury are known that can result in activation of podocytes and epithelial proliferation to form crescents.

### Type-I: Anti-GBM (Goodpasture syndrome)-

constitutes circulating antibodies IgG directed against the non-collagenous domain of alpha-3 chain of type IV collagen, present in the GBM and/or alveolar basement membrane. It accounts for 10%-15% of all diffuse crescentic GN and may present as GN alone or in combination with pulmonary hemorrhage (Goodpasture syndrome) and as ANCA associated “dual antibody disease”.^14^ Reported pediatric cases are very rare (n=31) with a higher prevalence in girls (M/F ratio 1:4).^15^

### Type-II: Immune complex mediated injury-

Multiple stimuli lead to crescentic proliferative GN including infections, systemic diseases, and preexisting primary GN. In most cases the serology and histology help diagnose the underlying disease, such as, presence of IgA deposits on IF in IgA nephropathy, antistreptococcal antibody positivity and sub epithelial deposits in PIGN, antinuclear antibodies (ANA) positivity and a “full house” (IgG, IgA, IgM, C3 and C1q) positivity on IF in LN. IC mediated RPGN is the most common and severe form, particularly in children with PIGN, IgA nephropathy/vasculitis and LN.^14^

### Type-III: Pauci-immune necrotizing and crescentic GN:

is characterized by few or no immune deposits on immunofluorescence and is usually associated with systemic vasculitis. ANCA associated vasculitis (AAV) is characterized by the destruction of small and medium-sized arterial vessels, in the presence of circulating autoantibodies toward the cytoplasmic region of the neutrophil (ANCA) predominantly against proteinase-3 (PR3) and myeloperoxidase (MPO). AAV includes granulomatosis with polyangitis (GPA), microscopic polyangitis (MPA) and eosinophilic granulomatosis with polyangitis (EGPA) and renal-limited vasculitis and drugs-associated ANCA positive GN. In AAV, these circulating autoantibodies (against PR3 or MPO) are found in 80-90% and are presumably produced in response to either respiratory infections or environmental factors. ANCAs induce activation of neutrophils resulting in release of inflammatory cytokines, reactive oxygen species and lytic enzymes and also initiate formation of neutrophil extracellular traps (NETs). These NETs along with inflammatory cytokines cause vasculitis affecting the respiratory tract and kidneys. ANCA positivity varies according to type of vasculitis, GPA usually shows anti-PR3 ANCA in 60–80% and produce a cytoplasmic pattern (c-ANCA). MPA, renal limited vasculitis and drug induced crescentic GN may show anti-MPO ANCA in 80–90% and produce perinuclear staining (p-ANCA). EGPA may also show anti-MPO ANCA in 35–40% of cases. Patients with MPA may have both types of ANCA. Ten percent of patients with GPA or MPA may have ANCA-negative GN limited to the kidneys and are taken under this spectrum with similar clinical features, biopsy findings and prognosis.^13^,^14^

The histological classification: focal (50% normal glomeruli), crescentic (>50% cellular crescents), sclerotic (>50% sclerotic glomeruli) and mixed (any other combination) has significant prognostic value. Validation studies have confirmed the predictive value of this system in adults and in children.^16^ In the pediatric study, the probability of having an estimated GFR of >60 ml/min/1.73 m2 at 2 years was 100% in the focal group, 56% in the crescentic/mixed group, and 0% in the sclerotic group. Most children present in adolescence with a female preponderance. In the setting of an inflammatory insult, PR-3 and MPO are translocated to the cell surface of neutrophils and monocytes where they interact with ANCA and stimulate neutrophils to undergo a respiratory burst and release their primary granule contents leading to tissue inflammation and endothelial damage.^17^

## CLINICAL MANIFESTATIONS OF RPGN

A nationwide RPGN survey from Japan showed that in children 0-18 years of age clinical manifestations included, proteinuria (60-72%) hematuria (68-83%) renal failure (average eGFR 41±34 ml/min/1.73m^2^ ) lung involvement (16%).^18^ RPGN in older children may present more commonly with complications like hypertensive emergencies, volume overload, pulmonary edema and cardiac failure.^14^

There may be features of underlying systemic diseases with skin (vasculitic rash), joint (arthritis), nervous system (convulsions and altered sensorium) and upper airway (sinusitis, septal perforation) /pulmonary involvement (hemoptysis and pulmonary hemorrhage).^11^,^14^,^19^

Anti-GBM RPGN is rare in children. Clinical manifestations may follow upper respiratory infection, with nonspecific symptoms or severe renal and pulmonary manifestations.

Renal involvement is a severe manifestation in ANCA associated RPGN (GPA, MPA), and can present as slowly progressive GN or AKI and often leads to ESKD, causing significant morbidity and mortality. MPA may present with isolated renal-limited form or overlap with polyarteritis nodosa, which may result in multi-organ involvement including renal, pulmonary, mesenteric, coronary artery and central nervous system. Renal involvement is seen in 94–100% of patients at onset of MPA and 50-100% in GPA but not in EGPA.^14^,^19^

## LABORATORY EVALUATION IN A CHILD WITH RPGN

All patients suspected with RPGN need investigations including serology and urgent biopsy.

- Urinalysis especially microscopy will show microscopic hematuria, RBC casts
- Renal function- always impaired in varying proportion.
- Serum electrolytes may be abnormal depending upon degree of AKI.
- Complete Blood Counts -anemia, neutrophilia and thrombocytosis are common.
- C-reactive protein (CRP)
- Spot urine protein to creatinine ratio may be elevated to nephrotic range (>3)
- Serologies-Anti-streptolysin O titer (ASOT)/anti-DNAase B (poststreptococcal glomerulonephritis), hepatitis B- virus surface antigen (HBsAg) and anti-HCV antibody
- Complement- C3, C4 (normal in Pauci-immune, anti-GBM disease and IgAN, low in PIGN (C3), lupus nephritis (C3, C4), CH50, anti-nuclear antibodies (ANA), anti-double-stranded DNA antibodies (anti-ds DNA) for lupus, serum IgA levels for IgAN, anti-GBM IgG antibodies, ANCA levels. ANCA screening is done by indirect immunofluorescence (IF) and ELISA for PR3 ANCA as first step and later IF for MPO.
- Radio-imaging: Chest X-Ray PA view, Echocardiography, Ultrasound kidneys and CT scan as indicated.
- Renal Biopsy: to confirm etiology (crescents in >50% of glomeruli), to identify underlying cause on IF to help classify patients into IC, PI and anti-GBM mediated RPGN (Table-II), to assess the degree of renal damage (percentage of glomeruli with crescents, type of crescents (cellular, fibrocellular and fibrous) to predict the reversibility of renal damage (activity and chronicity index, active proliferative with cellular crescent).^6^,^13^
- Genetic studies for idiopathic or primary MPGN, atypical HUS and should be done where facilities are available.

**Table II:** Clinical and Laboratory Features of Common Causes of RPGN.

Cause	Typical features	Serology	Complement	Biopsy	Outcome
Poststreptococcal glomerulonephritis	Sore throat/impetigo, gross hematuria, edema, oliguria, HTN	ASO, anti-DNase B antibodies	Low C3, normal C4	Granular immune deposits	Recovery in 2 weeks. No relapse
Lupus nephritis (diffuse proliferative, class IV)	SLE, gross hematuria, proteinuria, HTN	ANA, anti–dsDNA	Low C3, low C4	Granular immune deposits	Prolonged course, Relapses & remission. Mortality and ESKD
IgA disease	Persistent /episodic gross hematuria, proteinuria, HTN	Negative	Normal	Granular immune deposits	Varies with biopsy findings
Anti–GBM disease	Macroscopic hematuria and hemoptysis, acute kidney injury	Anti–GBM antibodies	Normal or increased	Linear staining for IgG and C3	With PLEX better outcome, No relapses high mortality if lung involvement.
AAV Microscopic polyangiitis Granulomatosis with polyangiitis (Wegener)	Non-specific, upper airway obstruction, arthralgia; hemoptysis, purpura, polyarthritis nodosa	p-ANCA c-ANCA	Normal or increased	Few/pauci-immune	Relapses may occur but less in MPA compared to Wegener, need long term follow up and immunosuppressive therapy

HTN-hypertension, ASO-anti-streptolysin O, ANA-antinuclear antibodies, anti–dsDNA- anti–double-stranded DNA, P-ANCA- perinuclear antineutrophil cytoplasmic antibodies, C-ANCA- cytoplasmic ANCA, SLE-systemic lupus erythromatosus,ESKD-end stage renal disease, PLEX-plasma exchange, **Adapted from:** Critical Care Nephrology, Chapter 47 2017.

## MANAGEMENT OF CHILDREN WITH RPGN

### A) Supportive Care:

All children with RPGN require strict monitoring for complications of AKI, respiratory support and underlying systemic disease management in the intensive care unit. Control of hypertension, dialysis for uremia to stabilize renal function along with electrolyte abnormalities, correction of anemia, treatment of intercurrent infection and care of nutrition are essential measures for any sick child with RPGN. Use of antiplatelet agents to prevent thrombotic complications associated with systemic vasculitis, prophylaxis for osteoporosis and gastric protection may be necessary in long term management. Decision for dialysis and renal biopsy as well as initiation of aggressive immunosuppressive therapy should be done on a priority basis.^6^,^13^

### B) Specific Treatment: (Algorithm 1):

Untreated, RPGN typically progresses to ESKD over a period of days, weeks or a few months. Patients with fewer crescents may have a slower more protracted course. Treatment regimens and management strategies have been extrapolated from adult studies due to limited experience in children.^19^,^20^ The European League against Rheumatism and European Renal Association-European Dialysis and Transplant association (EULAR-ERA EDTA) recommendations and the SHARE project findings suggest intravenous high dose steroids and cyclophosphamide (CYC) for 3–6 months as first-line therapy in children.^19^,^20^ Rituximab (RTX) is an alternative for children with refractory or relapsing disease.^19^,^20^ Mycophenolate mofetil (MMF) has also been used as induction therapy. Plasma exchange is an optional treatment in rapidly progressive renal failure or when alveolar hemorrhage is present.^20^,^21^ Given high risk of severe infection, infertility and secondary malignancy risk with CYC, alternative agents like MMF, or RTX and biological agents are being evaluated.

**Algorithm 1:**
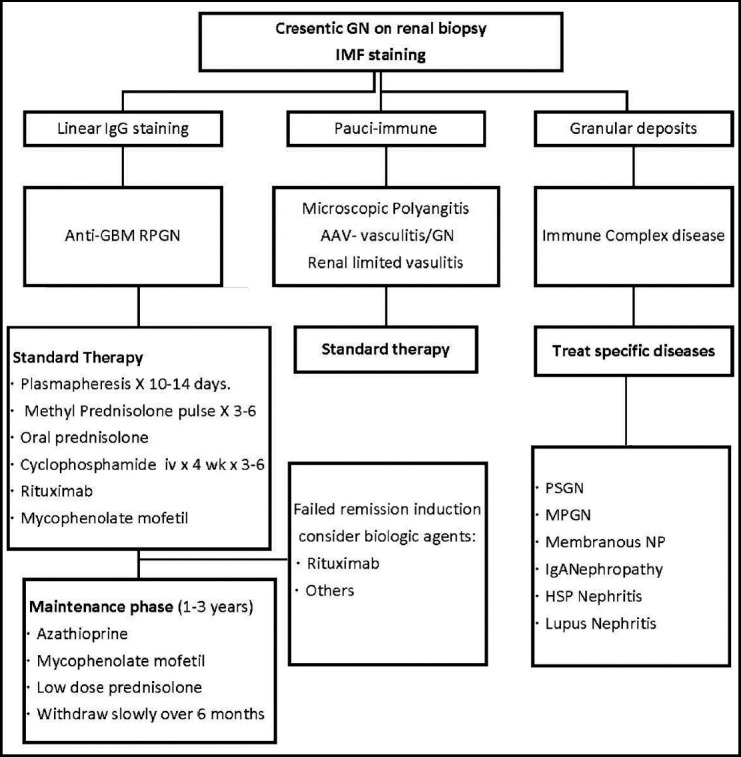
Treatment of Crescentic /Rapidly Progressive Glomerulonephritis

## INDUCTION OF REMISSION

### a) Standard Approach:

A combination of high dose glucocorticoids and either CYC or RTX and therapeutic plasma exchange are included in the initial treatment for remission induction.^14^,^20^

**Glucocorticoids** remain a cornerstone in remission induction and maintenance. The optimal dose, route, and duration of glucocorticoids remains uncertain. The common regimen used includes IV pulses of methylprednisolone (MP 15–30 mg/kg, maximum 1 g/day) for 3–6 days, followed by high-dose oral prednisone (1.5–2 mg/kg daily) for 4 weeks, with tapering to 0.5 mg/kg daily by three months and alternate day prednisone for 6–12 months.^1^

**Cyclophosphamide (CYC)** is an alkylating agent that causes broad immunosuppression and has been used widely for RPGN. Despite good response rates, the risk of relapse and toxicity has led to multiple studies comparing oral daily with IV pulse cyclophosphamide.

CYCLOPS an open label multicenter randomized controlled trial (RCT) in ANCA-associated vasculitis showed that IV CYC was non-inferior to oral CYC with respect to time to remission over 18 months and was associated with lower cumulative dose but higher relapse rates (50% higher than oral on follow up study).^21^,^22^ Oral dose of CYC varies from 2–3 mg/kg/day for 8– 12 weeks, with a maximum 150 mg/kg cumulative dosage and intravenously 500–750 mg/m^2^ mg/dose every two weeks with total 6 doses. The dose should be adjusted to maintain a leukocyte count of 3000–4000/cu mm or an absolute neutrophil count of > 1500.^14^,^19^,^23^

**Rituximab (RTX)** is a B-cell depleting anti CD20 monoclonal antibody found effective in induction of remission usually in cases of AAV with active crescents. RTX is preferred over CYC as first line remission induction therapy for patients in whom CYC is contraindicated or presents a risk of infertility.^22^ It appears to be a more promising therapy for AAV.^22^-^24^ The Rituximab in AAV (RAVE) trial (197 patients) compared rituximab with standard CYC and RITUXIVAS study (44 patients) compared addition of RTX in standard regimen (MP pulse, CYC) with standard regimen and found that RTX was as effective as CYC for induction of remission in newly diagnosed cases of AAV and appeared to be superior in patients with relapsing disease.^22^ High cost, availability of monitoring (CD19 levels) and relative risk of infections given prolonged immunosuppression are limiting factors for RTX use and CYC remains a cornerstone of therapy for many developing countries including Pakistan. RTX has been used as 375 mg/m^2^/week for 4 weeks with target being CD19 counts of less than 1%.^25^

Methotrexate (MTX) and MMF have also been studied for induction of remission. The trials NORAM and MYCYC showed that MTX and MMF may be used to induce remission in selected cases in whom conventional therapies have failed or are contraindicated and are at lower risk of relapse.^19^,^21^

**Plasmapheresis** (PLEX) has been used for the treatment of crescentic RPGN with variable success. The rationale of using PLEX is the rapid removal of antibodies which are involved in pathogenesis and to decrease the severity of vascular injury and end-organ damage. Therapeutic plasmapheresis is recommended for patients with pauci-immune crescentic GN, anti-GBM GN, and life-threatening pulmonary hemorrhage, and it might be beneficial for patients with refractory immune complex RPGN (due to SLE or severe proliferative GN).^20^,^21^,^26^ Intensive plasma exchanges for two weeks has been recommended for children with pulmonary hemorrhage requiring dialysis or with unsatisfactory response to induction treatment.

### PLEX in AAV:

The MEPEX trial showed reduced mortality and improved renal function in the PLEX arm (compared to IV MP and CYC) earlier on, however at four years there was no difference in ESKD and death.^27^ The PEXIVAS study reported that PLEX does not reduce the risk of ESKD or death in patients with severe AAV.^28^ The current evidence from PEXIVAS, MEPEX and other case series does not suggest a beneficial role of PLEX long term over standard of care in AAV. PLEX also incurs high cost and resources.

### PLEX in Anti -GBM disease:

Is the initial treatment of choice to remove circulating antibodies. Followed by immunosuppressive therapy with high dose MP and CYC or RTX to reduce antibody production.^27^ KDIGO guidelines for GN recommend PLEX with 60 ml/kg volume replacement daily in anti-GBM disease for 14 days or until anti-GBM antibodies are undetectable.^29^ Anti-GBM disease does not usually have a relapsing course. long-term maintenance therapy is not required however low dose prednisone, azathioprine (AZA), or MMF may be used as needed. Postponing transplantation for at least six months after antibodies have become undetectable reduces the recurrence rate significantly and is the accepted guideline. 30

## MAINTENANCE OF REMISSION

The maintenance therapy for RPGN based on consensus recommendation are low dose oral prednisolone, AZA, MTX, MMF and more recently RTX.^20^,^21^

### AAV:

In the maintenance phase, a combination of low-dose steroids and AZA, RTX or MMF can be used for an undefined follow-up period. The vast majority of patients with AAV will achieve remission with induction therapy, but about a third of them will relapse by 18 months and another third will remain in relapse-free remission for more than a decade.^19^,^21^

CYCAZAREM (CYC versus AZA for Early Remission phase of vasculitis) trial found no difference in relapse rates at 18 months.^31^ The IMPROVE trial (International MMF Protocol to Reduce Outbreaks of Vasculitides) found AZA to be superior to MMF for maintenance of remission.^32^ RTX has been increasingly used as maintenance therapy in AAV and is considered a safe and effective alternative to AZA.^21^ MAINRITSAN (Maintenance of Remission using Rituximab in Systemic ANCA-associated Vasculitis) showed sustained remission at month 28 with RTX compared to AZA.^33^

Duration of maintenance therapy (prednisolone and AZA) is usually for 18–24 months and may be withdrawn slowly over 6 months if patient has maintained remission for 12 months.^34^ This duration of therapy may be extended to 3-5 years in patients with either relapses or elevated ANCA titers.^1^

## TREATMENT OF FAILED INDUCTION

With adequate therapy, remission can be achieved in 60% of patients.^35^ Failure of induction of remission require more aggressive therapeutic agents depending upon which protocol already used, availability of resources and expertise. Multiple agents like MMF, MTX, RTX, PLEX, TNF alfa receptor blocker (etanercept), TNF monoclonal antibody (infliximab), cytotoxic T lymphocyte associated antigen 4 (CTLA-4) IgG (abatacept), human anti-CD52 monoclonal antibody (alemtuzumab), anti-IL-6 receptor human monoclonal antibody (tocilizumab), and anti-IL-5 human monoclonal antibody (mepolizumab) have been used with promising results in adults and children.^19^,^21^,^22^

## TREATMENT RELAPSING DISEASE

Relapse is defined as either a rapid rise in serum creatinine along with active urinary sediments and or glomerular crescents on follow-up renal biopsy. In addition, manifestation of new extra-renal symptoms or worsening of existing symptoms may occur in relapsing AAV. Treatment is to increase the dose of corticosteroids and a change in disease-modifying agent, after excluding non-compliance. Second or third-line therapeutic agents for induction and/or maintenance like MMF, MTX, anti-TNF agents, RTX or tocilizumab should be considered.^36^

## OUTCOME OF RPGN

The outcome is determined by the severity of renal failure at presentation, promptness of intervention and the underlying renal pathology. Overall long term prognosis has improved with aggressive combination immunosuppressive therapy. Majority (60-70%) show normal renal functions^1^. However, it depends on multiple factors and poor prognostic factors include nephrotic syndrome, AKI requiring dialysis, large fibrous crescents, and a high chronicity index.^31^,^35^

Most children with RPGN from PIGN have an excellent prognosis without any specific treatment and >90% regain normal renal function at short-term follow-up with a risk of CKD up to 31% in developing countries.^35^ The worst renal prognosis is seen with IC mediated GN and LN with ESKD at 54% and 29% respectively. Renal outcomes in patients with RPGN with >80% crescents, tubular atrophy and interstitial fibrosis are poor with >50% risk of ESKD.^37^ The mortality rate in Goodpasture’s is about 30% mainly due to severe pulmonary hemorrhage. Pediatric AAV is associated with a high relapse rate, longer maintenance therapy and significant organ damage. Identifying optimal strategies that balance the adverse events and cost of maintenance therapy with active disease morbidity and treatment are critical areas of research. The development of novel agents and optimization of existing therapeutics are evolving research areas in all types of vasculitis. Several agents targeting different components of immune /complement mediated or genetic GN are under investigation and may change the paradigm of future management of RPGN.

## CONCLUSION

RPGN that presents with gross hematuria, oliguria, hypertension, edema and AKI, though uncommon in children, is a management challenge and is limited to tertiary nephrology centers. Timely referral, diagnosis and urgent treatment are essential for optimal renal outcome. In our health set up, high dose methyl prednisolone and cyclophosphamide pulses are the initial therapy followed by low dose prednisolone and azathioprine or mycophenolate mofetil as maintenance therapy. Rituximab and plasmapheresis may be used in select cases. Long-term follow-up and monitoring for relapses, adverse events, renal function, hypertension, infections and growth is recommended for all children.
